# The phenomenology of psilocybin: transformative insights for research and clinical practice

**DOI:** 10.3389/fpsyg.2025.1455902

**Published:** 2025-04-25

**Authors:** Antonio Metastasio, Elisabeth Prevete, Sofia Venturini, Andrea Garofalo, Beatrice Cecconello, Nicola De Pisapia, Ornella Corazza

**Affiliations:** ^1^Department of Clinical, Pharmacological and Biological Sciences, College Lane, University of Hertfordshire, Hatfield, United Kingdom; ^2^Azienda USL Umbria 2, Terni, Italy; ^3^Department of Human Neurosciences, Sapienza University of Rome, Viale dell’Università, Rome, Italy; ^4^Department of Psychology and Cognitive Science, University of Trento, Rovereto, Italy

**Keywords:** phenomenology, psychedelics, psychopathology, psylocibin, lived experiences, qualitative approach

## Abstract

**Introduction:**

Considering the increasing evidence supporting psilocybin’s efficacy in therapeutic settings, it is essential to deepen our understanding of its subjective meanings and effects to enhance its integration into psychotherapy. Current knowledge is primarily based on psychometric assessments or unstructured personal reports, leaving a gap in the qualitative analysis of subjective psychedelic experiences and the resulting changes.

**Objective:**

This study aimed to describe the subjective psychedelic experience with psilocybin in a structured, objective, and non-judgmental way (*Epoche*), while exploring its potential clinical applications.

**Methods:**

A phenomenological qualitative approach, integrating interpretative phenomenological analysis (IPA) and the dynamic analysis (PHD) method, was used to analyze self-reported psilocybin experiences. Participants who met the inclusion criteria of being healthy adults and who had experienced psilocybin without any other substances were recruited through convenience sampling. Semi-structured interviews explored dimensions such as emotions, bodily sensations, perception of time and space, relationships, values, and enduring transformation. Data were analyzed using thematic coding.

**Results:**

Ten interviews were carried out with voluntary participants. All the interviewees reported enhanced emotional and interpersonal sensitivity, increased empathy, a deeper connection to others, and a heightened ability to resolve personal issues as well as long-lasting insights into their lives and values. Participants also showed profound changes in behavior, attitudes, and interests, indicative of the potential for psilocybin to catalyze significant personal growth and development.

**Conclusion:**

This study highlights the transformative potential of psilocybin experiences and their relevance to psychotherapeutic practices. By employing phenomenological methods, we offer a structured understanding of these states, which could be used in future to provide guidance for their integration into therapy by giving a better insight into psychedelic experience.

## Introduction

Over the last two decades, there has been an increasing scientific attention on the potential therapeutic benefits of psilocybin to treat several mental disorders, including depression and anxiety, particularly among patients with life-threatening conditions (Goldberg et al., 2020), major depressive disorders (Carhart-Harris et al., 2018; Carhart-Harris et al., 2016; Davis et al., 2021), treatment-resistant depression (Johnson and Griffiths, 2017; Roseman et al., 2018), and nicotine and alcohol use disorders (Bogenschutz et al., 2015; Hendricks, 2014; Johnson et al., 2014; Johnson and Griffiths, 2017).

Increasing reports suggest that the therapeutic effects of psychedelics are not solely due to neurobiological mechanisms (Vollenweider and Smallridge, 2022). Instead, the subjective psychological effects of the psychedelic experience have been shown to play a fundamental role in recovering from mental illness (Miceli McMillan and Fernandez, 2023; Yaden and Griffiths, 2021). Particular attention is paid to the so-called mystical experiences, whereas the presence and intensity of such experiences promote recovery (Yaden and Griffiths, 2021) and are associated with improved well-being in healthy subjects (McCulloch D. E. W. et al., 2022) and improvements in positive attitudes toward life, death acceptance, spiritual significance, and meaningfulness (Griffiths et al., 2016). Furthermore, benefits such as positive psychological and social outcomes, also found in healthy volunteers, have been associated with these mystical-type and ego-dissolution experiences and may be long-lasting (Doblin, 1991; Griffiths et al., 2008; Griffiths et al., 2006; Pahnke, 1969). As for the safety data, psilocybin can moderately increase blood pressure and produce derealization symptoms and hallucinations that typically resolve within 6 h and become less frequent after repeated administration (Johnson et al., 2019), showing no severe adverse reactions in clinical settings.

Qualitative studies have revealed that while patients may approach psilocybin treatment with skepticism, those undergoing therapy can develop trust in therapists, and the care received can be profoundly impactful. Moreover, the ability to navigate and surrender to the intense experiences induced by psilocybin plays a critical role in the therapeutic process, and this ability varies widely among individuals. The existing studies highlighted psilocybin’s impact on emotional, psychological, perceptual, and relational experiences (Agin-Liebes et al., 2024; Belser et al., 2017; Smith et al., 2022). Psilocybin facilitates deep emotional processing across contexts, helping individuals confront painful memories, foster self-compassion, and improve emotional regulation (Agin-Liebes et al., 2024). Participants often report transformative shifts in identity, characterized by reduced self-critical thought patterns, enhanced interconnectedness, and revised life priorities (Belser et al., 2017). Evidence highlights that the psilocybin experiences are also marked by vivid visuals, profound states, heightened embodiment, and music-enhanced emotional ranges from distress to bliss, promoting personal growth and fostering stronger relationships (Smith et al., 2022).

In this context, understanding the full range of experiences, including potential adverse effects, is crucial in offering new insights into how patients process emotions, confront unresolved trauma, or achieve new perspectives on their mental health challenges. Such knowledge can enhance the therapeutic alliance by helping clinicians tailor integration strategies post-session and align treatments with individual patient needs. Hence, this study proposes a phenomenological analysis of the experience induced by psilocybin to better understand the nature of its subjective component and the underlying mechanisms through which the substance facilitates lasting psychological and behavioral changes in healthy individuals.

## Methods

### Participants

Participants were recruited through convenience sampling. To facilitate recruitment, an online advertisement was shared on social media for 6 months (March–September 2024), inviting individuals who reported a meaningful psilocybin experience to participate in the study. Those interested were asked to provide a brief written summary of their experience for an initial screening. Inclusion criteria included (a) being healthy; (b) an age of 18 years and older; (c) having had an experience with psilocybin (both in clinical and non-clinical settings); (d) no other psychedelic substances or a mixture of substances; (e) being fluent in English language; and (f) providing informed written consent to participate to the study. Subjects who could not attend the interview online or undertake the study procedures were excluded from the investigation. Out of 34 respondents, 10 individuals met the inclusion criteria and were invited to attend an online interview. Participants with multiple significant experiences induced by psilocybin were instructed to focus on a single experience for the study. No incentives were provided for participation.

### Study procedures and data analysis

Interviews were always carried out in English since the subjects were either fluent in the language or native English speakers. The phenomenological unfolding, hermeneutic analysis, and dynamic analysis (PHD) method for psychotherapy introduced by Stanghellini (2019) was used to explore how participants described and made sense of their experience (Stanghellini, 2019). The PHD method integrates narrative inquiry and hermeneutic phenomenological analysis to achieve a comprehensive understanding of subjective experiences. These approaches, alongside interpretative phenomenological analysis (IPA), are well-established qualitative research methods, particularly valuable for investigating complex phenomena (Breeksema et al., 2024). This approach has been successfully applied in previous studies to investigate subjective experiences associated with substances such as synthetic cannabinoids (Corazza et al., 2020; Kassai et al., 2017) and psilocybin (Turton et al., 2014). Participants’ accounts were analyzed and compared across several pre-defined dimensions, including emotions; perceptions of body, time, and space; relationships with others; sense of self; values; and enduring impact or transformation, while adhering to the principle of ***Epoché*** (suspension of judgment). These categories were informed by evidence that emerged from previous research on meaningful experiences associated with psilocybin (Barrett et al., 2020; Griffiths et al., 2018; Jastrzebski and Bala, 2013; McCulloch D. E. et al., 2022; Smigielski et al., 2019; Wittmann et al., 2007). Subjects could describe the experience freely, allowing themes to emerge naturally alongside those identified *a priori*. All interviews were audio-recorded and transcribed. The study was approved by the University of Hertfordshire Health, Science, Engineering and Technology Ethics Committee (Approval Number: LMS/PGR/UH/04113).

## Results

### Sample

The sample included 10 participants from both clinical and non-clinical settings [mean age 32.3 years (range 25–36 years)], *F* = 2, who mostly reported their experience in different contexts, retrospectively. For more details, see Table 1.

**Table 1:** Lived experiences with psilocybin.

	P. 1	P. 2	P. 3	P. 4	P. 5	P. 6	P. 7	P. 8	P. 9	P. 10
Demographics	F, late 30s	M, 30	M, 30	M, late 30s	M, mid 30’s	M, early 30s	M, mid 20s	M, 31	M, 36	F, mid-30s
**Context of Use** (Setting, substance(s): dose, place, alone/with others)	Clinical setting (Experimental trial) Psylocibin – five capsules, precise dose: ND Kings College, London (United Kingdom): ND	Non-clinical setting (first experience in his early 20s) Psilocybin in the form of magic mushrooms, bought in a specialized shop — dose: ND Amsterdam (the Netherlands), in a park, in summer during the daytime With his girlfriend and his brother while a friend of his remained sober and looked over them	Non-clinical setting (experience in his early 20s) Psilocybin in the form of magic mushrooms — dose: ND Amsterdam (the Netherlands), in a park during the daytime With his brother and a friend	Non-clinical setting (experience he had before 3 years) Psilocybin in the form of dried mushrooms – 2.2 g from the mycelium he had previously bought online: ND Alone	Non-clinical setting (first experience he had many years earlier) Psilocybin in the form of magic mushrooms – dose: ND Amsterdam (the Netherlands), in a coffee shop and then in a park With his friend	Non-clinical setting (first experience in a legal recreational setting) Psilocybin in the form of truffles – dose: ND At home and then out for a walk in open air With the company of his brother and his friend	Non-clinical setting Psilocybin in the form of dried mushrooms — dose: ND Northern Italian city, at home with a friend	Non-clinical setting (first psilocybin experience) Psilocybin in the form of handpicked dried mushrooms – 1.2 g Northern Italian city, in the mountains; With three other people	Non-clinical setting Psilocybin in the form of mushrooms that he gathered – 1–1.5 g ND, at a friend’s house With five other friends	Non-clinical setting (first experience a few months earlier in a legal retreat) Psilocybin in the form of truffles – approximately 13.5 g (12 g initially and another 1.5 g after 1.5 h) of psilocybin Amsterdam (the Netherlands, ND)
Emotions	Mild anxiety associated with arousal due to positive expectation at the onset; the experience culminated reaching quasi-euphoric and entactogenic effects; psilocybin showed its capacity to facilitate deep emotional engagement and dynamic shifts, possibly promoting emotional processing	Going through a vast spectrum of emotions, starting from anxiety and fear of the unknown, then moving to discomfort culminating with euphoria, happiness, and well-being (the experience was claimed as *“the most beautiful experience”* of his life)	Initial emotions were surprise and awe that gradually transitioned toward a more positive one like excitement, which had quasi-euphoric and entactogenic effects.	Significant personal growth that helped him confront fears; The emotions were overall positive; there was a “*crescendo*” starting from dissatisfaction reaching an euphoric phase	An overall sensation described during the psychedelic experience of innocence, characterized by an absence of anxiety, fear, or any sense of danger	*“… I was feeling empathetic and naked ….”* These two emotions helped to blur the interpersonal boundaries, allowing to look at their real self without filters	The emotions changed quickly, from initial apprehension to eventual fascination and wonder, with an underlying tone of positivity and introspection throughout the experience. This had a positive impact and facilitated the positive effect that the session had on the individual	*“I was feeling very confident. While letting my emotions flow…”* The emotions changed significantly; he started from being calm and positive before the experience, then going through difficult and painful emotions associated with an important sense of embodiment with a resolution. Toward the end, he started feeling calmer and more tranquil	Very intense emotions; There is a passage through a phase described as vanishing consciousness (perhaps a form of ego dissolution) followed by empathy and connection with the other individuals. The emotions are therefore difficult to control, overwhelming but positive	Going through almost the entire spectrum of emotions, starting with fear and apprehension at the beginning of the experience that eventually morphed in curiosity
Lived body	Bodily sensations and experiences during the psilocybin session highlights the complex interplay between physical perceptions and psychological experiences. She went through a disembodied experience similar to out-of-the-body experiences, underscoring a disassociation from physical self-awareness and leading to an immersive and imaginative experience	The bodily experience also was a rollercoaster, spanning from unpleasant sensations starting with cold and hunger, then going through a phase where he could discern all the minute details of his skin *“like I was observing through a microscope”,* reaching subsequently a phase of wellbeing. What is remarkable is that despite experiencing his body sometimes like he *“was a child”* he never lost the contact with it	Initially, the experience was predominantly visual, with bodily sensations playing a minimal role; however, by the end, he experienced some fluctuations in temperature perception, with a distinct sensation of energy moving through the body. The visual experience, however, was overwhelming and prevented the subject from focusing on his body	The lived body experience was in parallel with the emotions. The body was immersed in positive feelings and sensations. This phenomenon was present both at visceral and superficial levels	No adverse physical reactions such as nausea or changes in body temperature during the experience were reported. Instead, they experienced a profound sense of their body disintegrating, as the usual boundaries delineating the self from the environment shifted and blurred. A deep sense of wonder was a hallmark of this experience	*“I could sense my body more deeply, some kind of energy was running through my spine and this made me think of the chakra theory”*	The session had a positive effect on the body with a gradual disappearance of pain and absence of muscular tension. Visual and auditory perception was somehow enhanced and transformed leading to the phenomenon he described as a sphere	*“I was able to localize a point of suffering in my abdomen*.” There has been an embodiment of the emotion and consequently the body went through different experiences going through pain and eventually calm and relaxation	Going through a primordial and probably preconscious feeling together with a sensation of physical wellbeing. The bodily sensations are very strong and thorough the proprioception are teaching the individual	The lived body is going through a complex self-experience (tingling sensation, feeling fluid and ethereal) that helped her gain self-awareness and to give a meaning to the experience when she reaches the conclusion that the body is the vessel of the journey
Lived time	The patient is living in the moment but retains an insight of the time passing. This temporalization seems to be an intra-festum experience (instantaneousness, feeling of enjoyment here-and-now, disjointed from past and future)	He experienced a profound sense of temporal fluidity, feeling as if time accelerated, with numerous events unfolding simultaneously	The person was focused on the present experience: *Hic et nunc*. There was a perception of the passing of time	During the peak intensity of their psychedelic journey, he found himself completely detached from the concept of time. This sense of timelessness was accompanied by a smooth, unimpeded flow of moments, where everything seemed to occur in perfect synchrony, without any friction or resistance	During the experience, he encountered a profound disorientation in their perception of time and space, finding these concepts to be inextricably linked and altered to the point of incomprehensibility. The temporalization is an intra-festum experience, characterized by a sense of instantaneousness, here-and-now sensation, in a present disjointed from both past and future. Salience of time is also lost	“*Every moment was deeper than usual…I had the perception of living many experiences in a short time*.” The lived time is altered; the individual describes it like if it has been compressed. The salience of the single experiences, despite being compressed, seems to be augmented	The experience of the lived time, as it happens frequently with psychedelics, changed its perception, and became less salient. Despite an initial sense of curiosity about the passage of time, as the participant begins to immerse himself in music and the psychedelic experience, time becomes irrelevant	Time dilated. There was no interest in looking at the time. The dose was not so strong. The lived time changed; somehow, it slowed down but also his salience disappeared so the individual was not concerned by it	The lived time was also affected during the experience; it was at first a slowdown that eventually culminated with a disintegration of the conception of time. This phenomenon is in tune with the overwhelming and deep experience that the individual describes	Lived time experience was not very affected during the session; there was no change in the perception of it
Lived space	She presented an extreme awareness of the space, which is also very realistic but at the same time she could change settings without any effort	The space becomes a continuum, and the world is a single fabric woven together without breaks or gaps	He experienced pareidolic illusion (the branches were arms moving in the air) associated with perception distortion where the familiar became unfamiliar. An unusually intense and vivid vision was the pivot of the experience	He describes a profound alteration in their perception of space, which took on a tangible quality. The perception of the lived space changed, and the person started interacting with the space itself and altering it through a dance	Lived space changed, it was perceived as something breathing and therefore “*alive*”, changing constantly and depriving the person of reference points. The participant recognized that our understanding of reality is deeply influenced by this dynamic interplay, suggesting a fluidity and malleability in how reality is constructed, experienced, and guided by the intertwined processes of thought and sensory perception	The non-ordinary perceptions he had were interpreted by the individual as a message about the ability to know the universe through a better understanding of his own consciousness	There was a distortion of the surrounding environment. This experience contributed to a sense of deep introspection on the complexity and profundity of life and a sense of wonder around the nature of the psychedelic experience	Nature had a big impact during the experience and colors were particularly intense. The lived space was perceived like brighter with more intense colors	*“The living room seemed to be breathing, and I could see a variety of geometric shapes with my eyes closed.”* The subject describes visual illusions and hallucinations. The room changed shapes and he felt as if it was breathing	The lived space was affected by the overwhelming intensity of the experience
Otherness	Her psilocybin journey underscores the complexity of interpersonal relationships	His experience of otherness was infused with deep emotions that lead to the establishment of a quasi-telepathic communication	*“I was happier like the other two” “minder.”* The participant and two companions shared a heightened sense of happiness during their experience; there was also an enhanced sense of empathy.	*“I had the experience alone, so I had no interaction with others”*	In the midst of their intense psychedelic journey, they engaged in brief, yet meaningful interactions with one another, sharing glimpses of their individual experiences	*“Ultimately, I realized that alternative means of communication, other than spoken language, were very important.”* The relationship with others evolves from contrast and resistance (at first after he shared with them the feeling of being naked) to a more emphatic one	The individual felt he was able to communicate with people far away using a sort of telepathy that consisted in communicating concepts but also feelings. Overall, the experience with others was a complex interplay between social interaction, rationalization, solitude, and sensory perception during the psychedelic journey	*“…I felt at ease with others. I questioned myself about knowing how to be alone and knowing how to be with others.”* The subject was aware of his emotions and was able to control them to avoid overflowing the other participants	Breaking interpersonal barriers, syntony, and empathy were the key elements of the relationship he had with the other co-sitters	The others were also going through a heavy and emotional experience. They were physically sick; however, despite the burden, they were also emanating positive sensations, safety, and tranquility. This allowed the establishment of a deep connection based on reciprocal trust, empathy, and affection
Selfness	There is a clear trajectory from the beginning where she is amazed and puzzled and at the end where all the experience is integrated and there is a clear message for herself and for her to deliver. Although the metamorphosis of experience involves profound changes in the perception of the Self	*“It was a deep spiritual experience. I know now that there is a greater force that runs the universe. If we let ourselves go with the flow, everything goes well*.” The experience revealed a profound spiritual awakening, that helped him improve his artistic skills to overcome mental blocks	There is a clear effect on the self with a switch from the self-referentiality to more openness and involvement in the external world, particularly nature	The experience affected the pre-reflexive and reflexive self. In the first case, the euphoric feelings were interacting with the body and in the second one he could feel an interaction between his reflective self and an external, ethereal entity	The overarching sensation throughout the psychedelic journey was one of profound amazement and awe, which encompassed all aspects of the experience	*“During the experience I was aware of my prejudices.”* There is a transition from a pre-reflexive self to a reflexive one when the subject develops a self-consciousness and wants to use it to better understand the experience and share it with others	It is possible to see in this description both the pre-and reflexive self; he can describe the communion with the environment and with others and the ability to communicate with them. He is also, however, to reflect on his concept of self and shifting from the pre-reflexive to the reflexive ones	*“The experience left a sign on me, but I wasn’t able to integrate it. I know I touched something important I wasn’t able to grasp and that I should investigate more in my life.”* The reflective self was unable to process the experience and the elaboration was somehow partial; there was an acknowledgment of positive feelings but also of suffering	*“I had some beautiful realizations, feeling an understanding of existence, as if a benevolent and peaceful force was present.” A*t first, only the pre-reflexive self was affected, with strong sensations, impossible to describe and to access consciously	The experience shows an evolution from a pre-reflexive self when she was in a dreamy state and was confronted by her past memories and experiences. This trajectory culminates with the phase of ego dissolution that was lived as an extraordinary event. She became more lucid, able to expand her mind because the synesthesia she described helped her make connections between different areas of the universe
**Values**	The psilocybin experience catalyzed a profound reassessment and reaffirmation of personal values, emphasizing a deeper connection with the environment, empathy toward all living beings, and a shift toward a more vegan lifestyle	*“I am still learning since then…This event changed my life.”* The experience provided a general enlightenment and systematization of beliefs and values that were already existing in him.	*“I started to doubt something… I became more open*.” The experience did not revolutionize the subject’s values but helped to make them more relative and therefore made him more open to new experiences	During the experience, the participant engaged in a profound reassessment of his core values, specifically focusing on courage, morality, and justice — qualities they felt were notably absent in society	Regarding values, the experience did not alter the participant’s rational conception of value, nor did it fundamentally change their ethical or moral viewpoints. This distinction underscores the psychedelic experience’s capacity to enhance emotional intelligence and empathy without necessarily shifting one’s core beliefs or ethical principles	The legacy of this experience were positive values such as more empathy, more awareness in the importance of communication, and more respect toward others	The individual seems to have gained a better relationship with nature; he starts believing that mushrooms, being natural, somehow unlock something that is already present, a “*natural feeling*.” Overall, the values expressed in this experience revolve around naturalness, authenticity, connection with nature, and the enduring impact of the psychedelic experience	The values were positive ones: he was more connected with himself, the others, and nature	The values are positive with a strong emphasis on sharing and empathy	As often described during non-ordinary state of consciousness events, the previous values were enhanced; in this case, the person became more connected with nature. She also developed a new attention for life in all its forms and became more mindful and spiritual
Enduring impact/transformation	The reflections post-psilocybin experience revealed significant shifts in awareness, values, and outlook on life. An increased recognition of the environment and a newfound appreciation for life’s subtleties underscore the deepening of their mindfulness and connection to the present	*“I have a deeper understanding about myself, and I feel more open as a person and as an actor”*	There was a profound personal transformation, marked by increased sensitivity and humility. He lost his previous certainties, but this event was the catalyst for creativity having a notable impact on his lifestyle	The psychedelic journey led to a significantly deeper connection with their innermost feelings and needs, enhancing their ability to engage with their super-ego — the part of the psyche responsible for critical self-evaluation and moral standards — with greater calmness	This initial psychedelic experience marked a pivotal shift in his perception of reality, impacting him on both psychopathological and philosophical/metaphysical levels	*“I was able to see through the formal aspects of people’s personality and to take them more lightly”*	Overall, the described experience suggests the potential for enduring transformation through enhanced self-awareness and a shift in perception	The individual became more in tune with his own emotions	“*This experience has left me with an acceptance of death. My hypochondria vanished, and I’ve been left in a state of physical and mental well-being that was very optimistic and positive, making me feel prepared for anything that might happen*”	The experience was very transformative. The person had a different, clearer outlook of the world. She became more drawn toward nature, more emphatic. She began owning a new and deeper form of knowledge but was aware that other individuals might not possess it, rendering difficult to communicate it and leading to a feeling of solitude that was felt as bittersweet. The impact was anyway deep and long-lasting

P, participant; F, women; M, men.

### Lived experiences of psilocybin

All details related to the lived experiences following psilocybin use were analyzed considering the main phenomenological dimensions including emotions, body, time, space, others, self, values, and enduring transformation and impact according to the PHD method. The results have been summarized in Table 1.

### Overarching insights across psilocybin experiences

#### Context

The contexts in which psilocybin was consumed varied widely among participants, reflecting a spectrum of environmental and social settings. These included controlled clinical environments, structured legal retreats, and recreational use in natural settings such as parks or mountains and private, domestic spaces like homes. Experiences spanned solitary journeys and group settings with friends or family, providing insights into the personal and shared aspects of these experiences.

#### Emotions

A wide range of emotional experiences were consistently described across all participants, beginning with apprehension, fear, or anxiety, which often transitioned into euphoria, awe, and profound happiness. As one participant described, “*I was apprehensive at first and very excited to take part*” (participant 1). While the emotional spectrum varied, the transformative arc of emotions was common, culminating in positive states like peace, joy, and empathy, with one participant reporting feeling “*empathetic and naked*” (participant 6). Emotional intensity was universal, with laughter, tears, and catharsis frequently noted, with one participant reporting that “*the emotions were very strong and led to important physical reactions like laughter and tears*” (participant 10) or another one describing that “*the emotions were very intense, feeling unmediated and primordial*” (participant 9). However, some participants found emotions overwhelming, while others experienced tranquility and emotional integration.

#### Lived body

A strong interplay between physical sensations and psychological states emerged. Many participants reported heightened bodily awareness, often described as energy flows, tingling sensations, or the perception of disintegration and merging with the environment. One participant reported “*a disembodied experience similar to out-of-the-body experiences*” (participant 1), while another subject described “*a profound sense of my body disintegrating, as the usual boundaries delineating the self from the environment shifted and blurred*” (participant 5). On the other hand, others noted fluctuations in body temperature, physical comfort, or the ability to localize pain and embody emotions. A recurring theme was a newfound connection to the body as a vessel for the psychedelic journey. For instance, one participant reported: “*I could sense my body more deeply, some kind of energy was running through my spine*” (participant 6), or another subject describing “*“I had a sense of physical well-being…I got the impression that the substance has something to teach me … through sensations*” (participant 9).

#### Lived time

Time perception was consistently altered and appears to be a central feature of psilocybin experiences. A majority of the participants described a distortion or complete loss of temporal awareness of time. They claimed that “*Time was not relevant*” (participant 1) or “*Time did not matter*” (participants 2 and 3).

Time often felt compressed, expanded, or irrelevant, creating a profound “here-and-now” sensation. Another participant described, “*I spent five hours in continuous becoming. I felt time was going fast, and many events were happening together, although it went fast, I enjoyed every single moment*” (participant 2). However, some participants experienced fluidity, with events unfolding rapidly, while others felt time slowed or became suspended, as noted by one participant, “*living that experience felt like a lot of time, more than any other moment*” (participant 8).

#### Lived space

Similarly, space perception was consistently altered. Participants described vivid and intensified colors, pareidolic illusions, and spaces appearing “alive” or “breathing.” Specifically, one subject remarked, “*I perceived the tree as being alive… everything was seen in a different way, with colors more intense and enhanced*” (participant 3). Nature played a significant role, often described as vibrant, interconnected, and conscious. For instance, a participant claimed that “*space became more alive; the world was inhabited by consciousness and unknown forces*” (participant 6). For some, space became continuous and immersive, as captured in the description, “*land and sky were fused together*” (participant 2). However, others noted distortions and an ability to reshape or interact with their surroundings, highlighting the dynamic and malleable nature of spatial perception during the experience.

#### Otherness

Social interactions and perceptions of others were also profoundly affected. Participants often experienced heightened empathy, deep emotional bonds, and alternative means of communication, such as telepathic-like exchanges. As one participant shared, “*I could see the others better; there were few phrases but loaded with emotions. I felt that language was unnecessary to communicate with others*” (participant 2). However, the degree of interpersonal focus varied, whereas some participants emphasized solitude or introspection, while others highlighted shared emotional experiences, with one remarking, “*we remained in synchrony throughout the experience*” (participant 9).

#### Selfness

On the other hand, the perception of the self underwent significant transformations, often involving a shift from pre-reflexive to reflexive states. Participants frequently described ego dissolution, spiritual awakenings, and profound realizations about their place in the universe. As one participant described, “*I experienced a deep acceptance of the surrounding reality and of myself, as if I had made peace with my inner demons*” (participant 9). This was often accompanied by a deep acceptance of oneself and an expanded sense of identity, moving from self-referentiality to openness and interconnectedness, as reflected in the account, “*it was a deep spiritual experience. I know now that there is a greater force that runs the universe. If we let ourselves go with the flow, everything goes well*” (participant 2).

#### Values

Psilocybin experiences often led participants to either reevaluate or reaffirm their core values. Many described gaining a deeper sense of empathy, a stronger connection to nature, and a move away from materialistic or superficial concerns. As one participant noted, “*I became less materialistic. I also learned the values of others*” (participant 2). For some, these experiences reinforced values they already held, such as a focus on relationships, spirituality, or living authentically, as reflected in the remark, “*I developed more empathy, awareness in communication, and more respect toward others*” (participant 6). For others, the shifts were more profound, challenging their previous ways of thinking and sparking significant personal change. One participant reported a shift toward a vegan lifestyle (participant 1). Moreover, participants often spoke about these moments as deeply meaningful, sometimes even life-defining, and they frequently emphasized the importance of integrating these insights into their daily lives. These experiences were described as both challenging and rewarding, offering clarity and a renewed sense of purpose.

#### Enduring impact

The enduring impact of psilocybin experiences was generally positive, with participants reporting lasting changes in self-awareness, creativity, relationships, and worldview, with one subject reporting “*I have a deeper understanding about myself, and I feel more open as a person and as an actor*” (participant 2). Common themes included increased mindfulness, greater tolerance and patience, and an improved ability to handle life’s challenges. As one participant noted, “*I became more understanding, I widened the confines of my interior self, and I opened up to new genres of music. I also developed more patience and more availability to listen to others*” (participant 6). Additionally, for some, the experience marked a profound spiritual or philosophical turning point, while others noted a subtler, yet meaningful, personal growth. This is highlighted by the reflection, “*before this experience, I did not fully understand myself or what could be felt and perceived. I realized that the people around me loved me, and I became much more tolerant with people*” (participant 7).

## Discussion and conclusion

Our phenomenological analysis shows that psilocybin induces profound shifts in perception, emotional processing, and self-awareness, holding significant implications for psychotherapy and the broader understanding of psychedelic-induced states. In general, participants reported a greater capacity to accept their emotions. These effects are supported by neuroimaging studies, demonstrating that psilocybin reduces amygdala reactivity to negative stimuli and that this attenuation relates to an increase of positive mood in healthy participants (Kraehenmann et al., 2015; Preller and Vollenweider, 2018). Interpersonal sensitivity, empathy, and connection to others were also enhanced, and participants seemed to gain insights into their lives and the ability to resolve personal issues often becoming more open and being able to face them from different perspectives. This aligns with previous research by MacLean et al. (2011), who showed that openness remained significantly elevated for 1 year after the psilocybin session in participants who had a mystical experience (MacLean et al., 2011). Similarly, subjects seemed to gain insights into their values, and the nature of reality, which is also in line with previous research showing that psilocybin induces subjective positive changes in attitude and personality (Studerus et al., 2012). These findings are in tune with similar research and confirm the effect that psilocybin has in healthy individuals.

Moreover, the dissolution of ego boundaries was often highlighted, aligning with the concept of “ego death” reported in psychedelic literature (Stoliker et al., 2022). This experience can be therapeutic, allowing individuals to transcend their self-centered perspective, reduce ego-driven behaviors, and develop a more integrated sense of self. These experiences also led to long-lasting changes in behavior, attitudes, and interests, enhancing emotional awareness even after the effects of the substance vanished, and highlighting the potential of psilocybin to catalyze personal growth and development. The ability of psilocybin to induce these profound experiences supports its use for therapeutic applications. By facilitating a temporary loosening of the ego, psilocybin can enable individuals to confront and reprocess traumatic memories or entrenched negative beliefs from a new perspective (Letheby and Gerrans, 2017; Mason et al., 2020), which was also noted in this study. One of the participants described suffering but in a relaxed manner, which highlights the potential psilocybin holds to promote positive emotional responses. This enhanced emotional sensitivity and empathy may improve social connectedness and resolve, key factors in many mental health issues, including depression and anxiety (Morrison et al., 2019; Taylor et al., 2020). Moreover, the insights gained during these experiences can provide individuals with new meanings and directions in life, potentially alleviating existential anxieties and providing motivation for personal change. While some participants reported a range of negative experiences, no significant adverse reactions were reported, as these negative effects were generally short-lived and resolved as the experience unfolded. Negative experiences included transient fear, anxiety, and apprehension at the onset, physical discomfort, such as changes in body temperature or nausea, disorientation in time and space, and feelings of emotional vulnerability. Moreover, as previously suggested, no serious adverse effect was reported due to limited sample size. However, we assessed a heterogeneous group, comprising both men and women and experiences from recreational and clinical use, which suggests the need to replicate such a study in a bigger sample for a better understanding of the generalizability of these results.

This study, while explorative and preliminary, demonstrates the significant benefits of employing a PHD phenomenological analysis of psilocybin experiences. Firstly, it provides novel insights into the subjective nature of consciousness alteration, enhancing our understanding of this complex phenomenon. Secondly, it identifies therapeutic elements inherent in psychedelic experiences, offering valuable guidance for integrating these elements into psychotherapeutic practices. Unlike previous studies that utilized a phenomenological approach but lacked methodologically guided qualitative data collection (Horvath, 2018; Houot, 2021; Miceli McMillan and Fernandez, 2023; Szabo et al., 2014; Szummer et al., 2017), this research addresses this gap by using semi-structured interviews and structured assessments. Additionally, it underscores the importance of a supportive and safe environment during psychedelic sessions, highlighting the critical role of set and setting in shaping the experience.

This research advocates for expanding the phenomenological assessment to include different psychedelic substances and non-ordinary states not induced by psychedelics. Through the exploration of categorically defined experiences, it offers a deeper understanding of altered states of consciousness (ASCs) under the influence of psilocybin and the enduring changes associated with it. Overall, these findings hold practical implications for clinical settings, as they can support clinicians in gaining a more comprehensive understanding of their patients and establishing stronger therapeutic relationships, while addressing the complexities of human psychology with greater precision and care.

## Limitations

This article is exploratory in nature, and we are therefore aware that the analysis does not fully meet the standards of high-quality phenomenological research (Nizza et al., 2021). We are, however, living the dawn of phenomenology applied to psychedelic research; therefore we need, in this phase, a pragmatic approach to collect as many cases as possible to build a database and to learn so that future qualitative research will fulfill high-quality standards. The main limitation of this study is that participants were asked to recall an experience that may have occurred years prior to the interview, possibly challenging the accuracy and reliability of their memory. Additionally, the study lacks a detailed assessment of adverse effects and precise dosage reporting, which further limits the robustness of the findings. This omission makes it difficult to draw a clear picture of psilocybin’s effects, given that the strength and nature of its impact are closely correlated with dosage. Variation in doses, or the lack of precise dosage data, may have significantly influenced the clinical and psychological outcomes reported by participants. The authors acknowledge that while small-scale studies provide valuable preliminary data, the findings are based on a limited sample size and may not be generalizable to a larger, more diverse population, thereby limiting the external validity of our findings.

## Data Availability

The raw data supporting the conclusions of this article will be made available by the authors, without undue reservation.
